# Multi‐Fluid MHD Simulations of Europa's Plasma Interaction: Effects of Variation in Europa's Atmosphere

**DOI:** 10.1029/2022JA030569

**Published:** 2022-09-09

**Authors:** Camilla D. K. Harris, Xianzhe Jia, James A. Slavin

**Affiliations:** ^1^ Department of Climate and Space Sciences and Engineering University of Michigan Ann Arbor MI USA

**Keywords:** Europa, multi‐fluid magnetohydrodynamics, Moon‐magnetosphere interactions

## Abstract

Europa's plasma interaction is inextricably coupled to its O_2_ atmosphere by the chemical processes that generate plasma from the atmosphere and the sputtering of magnetospheric plasma against Europa's ice to generate O_2_. Observations of Europa's atmosphere admit a range of possible densities and spatial distributions (Hall et al., 1998, https://doi.org/10.1086/305604). To better understand this system, we must characterize how different possible configurations of the atmosphere affect the 3D magnetic fields and bulk plasma properties near Europa. To accomplish this, we conducted a parameter study using a multi‐fluid magnetohydrodynamic model for Europa's plasma interaction (Harris et al., 2021, https://doi.org/10.1029/2020ja028888). We varied parameters of Europa's atmosphere, as well as the conditions of Jupiter's magnetosphere, over 18 simulations. As the scale height and density of Europa's atmosphere increase, the extent and density of the ionosphere increase as well, generating strong magnetic fields that shield Europa's surface from impinging plasma on the trailing hemisphere. We also calculate the precipitation rate of magnetospheric plasma onto Europa's surface. As the O_2_ column density increased from (1–2.5) × 10^14^ cm^−2^, the precipitation rate decreased sharply then leveled off at 2 × 10^24^ ions/s for simulations with low magnetospheric plasma density and 6.4 × 10^24^ ions/s for simulations with high magnetospheric plasma density. These results indicate that the coupling between Europa's plasma populations and its atmosphere leads to feedback that limits increases in the ionosphere density.

## Key Points

- A range of Europa's possible atmospheric profiles were explored in magnetohydrodynamic simulations to quantify their impacts on the plasma interaction
- Growth of Europa's ionospheric density is self‐limited by the coupling between Europa's atmosphere and the surrounding plasma populations
- Properties of Jupiter's thermal plasma precipitating onto Europa's surface vary strongly in response to Europa's atmospheric conditions

## 1 Introduction

Europa's plasma interaction is coupled to its atmosphere through various physical processes that transfer mass and energy between the atmosphere, Europa's surface, the ambient thermal plasma and energetic charged particles of Jupiter's inner magnetosphere, and the cold plasma of Europa's ionosphere. At Europa the O_2_‐dominated atmosphere is generated primarily by sputtering interactions between magnetospheric particles and the icy surface (Johnson et al., 2009). Above the surface, neutral atmospheric O_2_ is then ionized by solar photons and magnetospheric electrons, generating cold ionospheric plasma (Kliore et al., 1997). Ions can recombine with electrons to form neutrals, and ionospheric ions can undergo charge exchange with the atmosphere.

Though these individual physical processes are relatively well understood, the complexity of the system, potential variability in different components, and limited in situ observations make quantifying the coupling between Europa's plasma interaction and atmosphere challenging. Observations have constrained the column density of Europa's atmosphere to a range that nevertheless admits very different configurations. Furthermore, the density of Europa's atmosphere may vary due to variations in the populations of magnetospheric ions and electrons that generate atmospheric O_2_ through sputtering interactions with Europa's surface. The atmosphere comprises the source population for Europa's ionospheric plasma, and the ionosphere plays a critical role in shaping the electromagnetic fields resulting from Europa's plasma interaction. Therefore, the potential variations of Europa's atmosphere must be accounted for to characterize Europa's plasma interaction.

### 1.1 Potential Variability of Europa's Atmosphere

Remote observations of Europa's oxygen aurora constrain the column density of the atmosphere to (2.4–20) × 10^14^ cm^−2^ (Hall et al., 1995, 1998). Assuming a hydrostatic structure, the column density can then be decomposed into two parameters: surface density and scale height. However, neither of these parameters have been definitively constrained independently. Roth et al. (2016) estimated the scale height to be ∼100 km based on Hubble Space Telescope (HST) observations, while Monte Carlo models for the atmosphere have predicted scale heights as low as 20 km (see, e.g., Cassidy et al., 2007, and others cited in the recent review by Plainaki et al. (2018)).

Various models for Europa's atmosphere have attributed a significant fraction of atmospheric O_2_ to sputtering by thermal plasma (e.g., Cassidy et al., 2013; Vorburger & Wurz, 2018). Therefore, we would expect the density of Europa's atmosphere to increase when Europa is located at the center of Jupiter's dense plasma sheet (Bagenal et al., 2015), or when the global state of Jupiter's magnetosphere is such that the density of the plasma at Europa's orbit is elevated, both of which would cause the precipitation of thermal plasma onto Europa's surface to increase (Harris et al., 2021). The latter may occur in connection with increased volcanic activity at the inner Galilean moon Io, which provides the primary source of thermal plasma in Jupiter's magnetosphere (Bagenal & Dols, 2020; Yoshioka et al., 2018).

However, most of the O_2_ in Europa's atmosphere is likely generated in sputtering interactions between energetic ions and electrons and Europa's icy surface (Johnson et al., 2009). Jupiter's magnetosphere is populated with energetic charged particles with energies ranging from several keV to tens of MeV (Paranicas et al., 2009). Energetic particle populations in Jupiter's magnetosphere have been shown to change on timescales of years and decades (see, e.g., the depletion in ring current ion populations during the *Galileo* mission era discussed by Mauk et al. (2004)). In addition, energetic particle injections can cause short‐time‐scale variations, as recently observed by the *Juno* spacecraft and reported by Mauk et al. (2020).

Therefore, we anticipate that the populations of sputtering particles at Europa, including thermal ions and energetic electrons and ions, will be variable in time, and can cause the density of Europa's atmosphere to vary. Other changes in the atmosphere density could be caused by diurnal effects (Oza et al., 2019; Plainaki et al., 2013) or potentially by water plumes (Jia et al., 2018; Paganini et al., 2019; Roth et al., 2014; Sparks et al., 2016).

### 1.2 Representations of Europa's Atmosphere in Models for the Plasma Interaction

In the last few decades numerous computational models have been developed to simulate Europa's plasma interaction. Here, we review several examples to trace how the representation of Europa's atmosphere in plasma models has developed over time.

Saur et al. (1998) developed a fluid model for the plasma interaction that balanced the mass exchanged between Europa's atmosphere and ionosphere through ionization and recombination processes in uniform magnetic fields. They assumed a scale height of 150 km and varied the density of Europa's atmosphere, and calculated the rates at which mass was added and lost from the simulated atmosphere. They found that the most balanced state was achieved with a column density of 5 × 10^14^ cm^−2^. Saur et al. (1998) then calculated properties of the ionosphere, such as electron density, currents, and conductance, for this mass‐balanced case.

Schilling et al. (2007, 2008) used a single‐fluid magnetohydrodynamic (MHD) model to self‐consistently simulate the electromagnetic fields and plasma properties of Europa's plasma interaction, including Europa's induced field. They used a hydrostatic model for the atmosphere with a large scale height of 145 km and a surface density of 1.7 × 10^7^ cm^−3^; these parameters were chosen so that the atmosphere column density was that identified by Saur et al. (1998). They also included an asymmetric enhancement of the atmosphere density on the upstream side based on analysis of sputtering fluxes by Pospieszalska and Johnson (1989). The model also included the production of new ions by ionization, and the loss of ions by dissociative recombination.

Lipatov et al. (2010, 2013) implemented a hybrid modeling approach and identified the effects of different possible compositions of the magnetospheric ions on the plasma interaction. In the model atmosphere they incorporated contemporary modeling results by using two populations: cold O_2_ with a scale height of 200 km and thermal O_2_ with a scale height of 30 km (Cassidy et al., 2007). As in previous models, here the authors included production terms to calculate the rate at which new ions were added by photoionization and electron impact ionization. Due to the representation of multiple populations of kinetic ions in the model (O^++^ and S^++^ representing magnetospheric plasma, and cold and thermal O_2_^+^ pick‐up ions), the authors implemented different ionization rates for the different ions.

Rubin et al. (2015) introduced a two‐ion‐fluid multi‐fluid MHD model for Europa's plasma interaction which incorporated the effects of interactions between the neutral atmosphere and the plasma populations of Jupiter's magnetosphere and Europa's ionosphere. Their implementation of the atmosphere synthesized the innovations described above. The authors modeled Europa's atmosphere with an enhancement on the upstream side and included two populations to represent cold and thermal O_2_. They also included a comprehensive set of source and loss terms to model the effects of interactions between the plasma and the atmosphere on the model ion fluids. The model used one fluid to represent O^+^, including both the thermal magnetospheric ions and cold ionospheric O^+^ generated from the atmosphere, and a second fluid to represent cold ionospheric O_2_^+^. By this use of multi‐fluid MHD, including a separate equation for the electron pressure, they were able to calculate the spatially dependent production and loss rates separately for each fluid. In addition to mass, this model included source and loss terms for the momentum and pressure of the two ion fluids.

Several models have considered the effects of potential water plumes, modeled as localized enhancements of atmosphere density, on the plasma interaction. Blöcker et al. (2016) developed a single‐fluid MHD model for the plasma interaction and showed the effects of atmospheric inhomogeneities on Europa's Alfvén wings. Jia et al. (2018) added a plume feature to the atmosphere in the model of Rubin et al. (2015) to demonstrate how a plume could explain the magnetometer observations of the E12 *Galileo* flyby. Arnold et al. (2019, 2020) implemented a hybrid model for the plasma interaction, and used a similar atmosphere‐with‐plume configuration as Jia et al. (2018) to first model the E26 *Galileo* flyby and subsequently to simulate magnetic field signatures along a generic satellite flyby that passed through the plume.

Most recently, Harris et al. (2021) developed a multi‐fluid MHD model for the plasma interaction based on that of Rubin et al. (2015). They extended the model from two to three ion fluids, with separate fluids to each represent the magnetospheric plasma, O_2_^+^ generated from the atmosphere, and O^+^ generated from the atmosphere. The comprehensive coupling between Europa's atmosphere and the MHD fluids was retained from the model of Rubin et al. (2015). The authors used the model to conduct a parameter study characterizing the precipitation rate of magnetospheric plasma at Europa and its dependence on magnetospheric conditions. The precipitation rate was found to increase with the magnetospheric plasma density due to the generation of Europa's ionosphere from the neutral atmosphere by magnetospheric electrons. However, the effects of changes in the neutral atmosphere were beyond the scope of that study.

In recent years models for Europa's plasma interaction have prescribed atmospheres with a wide range of parameters, employing different surface densities and scale heights as well as different degrees of asymmetry between the trailing and leading hemispheres to account for increased precipitation of magnetospheric particles on the trailing/upstream hemisphere. The result is that between these different plasma models the atmosphere density may vary by an order of magnitude or more at the same location. For example, the density of O_2_ at the apex of Europa's trailing hemisphere, including the sputtering enhancement, is 1.5 × 10^9^ cm^−3^ in the simulations presented by Rubin et al. (2015), 1.2 × 10^8^ cm^−3^ in the simulation of Jia et al. (2018), 1 × 10^8^ cm^−3^, and 1 × 10^9^ cm^−3^ in the two cases presented by Arnold et al. (2019), and 7.5 × 10^7^ cm^−3^ in the simulations presented in Harris et al. (2021).

The representations of Europa's atmosphere in these simulations were all realistic given the current state of observations and modeling of Europa's atmosphere. Furthermore, all of these models produced reasonable simulations of the plasma interaction despite these differences in the modeled density and scale height of Europa's atmosphere. This indicates that the present observational constraints on Europa's plasma interaction admit a range in the atmosphere parameters. Therefore, in this study we have used the model of Harris et al. (2021) to vary the parameters of Europa's atmosphere through the parameter space bounded by the current observational constraints (Hall et al., 1995, 1998; Roth et al., 2016) to better understand how plausible variations in the density and scale height of Europa's atmosphere affect the plasma interaction.

### 1.3 Outline

Here, we extend the study of Harris et al. (2021) by conducting a parameter study of several simulations of Europa's plasma interaction, bounded by the existing observations of the atmosphere. In Section 2, we describe the setup of the simulations and the parameters that were varied for the study. In Section 3, we present and compare the results from each simulation, and we identify how different configurations for Europa's atmosphere affect the structure of Europa's ionosphere. In Section 4, we identify how Europa's atmosphere affects the spatial distribution and total amount of precipitation of thermal plasma from Jupiter's magnetosphere onto Europa's surface. In Section 5, we review our conclusions and caveats for this study, as well as future lines of investigation.

## 2 Methods

To better understand how changes in Europa's atmosphere affect the plasma interaction we conducted several simulations using the multi‐fluid MHD model presented in Harris et al. (2021) to span the parameter space of reasonable atmosphere variation for Europa. Here, we briefly summarize the model, highlight the updates we have made, and describe the setup for the parameter study.

The multi‐fluid MHD model for Europa's plasma interaction is based on the BATS‐R‐US MHD code (Toth et al., 2012) and was first applied to Europa in a two‐ion‐fluid version (Jia et al., 2018; Rubin et al., 2015). Later, Harris et al. (2021) extended the model by solving the multi‐fluid MHD equations for three ion fluids representing thermal magnetospheric ions, ionospheric O^+^, and ionospheric O_2_^+^, as well as the electron pressure equation for thermal electrons. Source terms in the mass, momentum, and pressure equations account for the effects of electron impact ionization, photoionization, recombination, and charge‐exchange on each fluid. Rubin et al. (2015) and Harris et al. (2021) give detailed explanations of how the occurrence rates of these processes are calculated and how their effects are implemented. In general, these source terms enable the model to simulate the coupling between the MHD fluids and the prescribed O_2_ atmosphere.

The equations are solved on a spherical grid with the inner boundary representing Europa's surface at *R* = 1 *R*_Eu_ (*R*_Eu_ = 1,560 km is Europa's radius), and extending to *R* = 128 *R*_Eu_ so that MHD waves reflecting from the outer boundary due to the sub‐Alfvénic nature of the magnetospheric flow would have minimal effects on the plasma interaction near Europa. At Europa's surface model, boundary conditions specified the interaction of the surface with the surrounding plasma. The EPhiO coordinate system is used to organize the simulation, with magnetospheric plasma flow along the *X* coordinate, the *Y* coordinate pointing toward Jupiter, and the *Z* coordinate pointing north, anti‐parallel to the Jovian magnetospheric magnetic field. Sources of mass, momentum, and pressure modify the ion and electron fluids to model the effects of electron impact ionization, photoionization, recombination, and charge exchange. These source terms are crucial for generating the ionosphere and accurately modeling the interaction between the ion fluids, electrons, and atmosphere. The 3D density distribution of Europa's O_2_ atmosphere is prescribed in the simulation, and the implementation of the atmosphere for this parameter study is described in more detail in Section 2.1. All of these features are described in more detail by Harris et al. (2021).

We have since updated the model's treatment of the magnetospheric plasma. In the simulations presented in Harris et al. (2021), one MHD fluid was used to represent the thermal O^+^ plasma of Jupiter's magnetosphere. However, the composition of Jupiter's magnetospheric plasma includes not only O^+^ but also H^+^ and S^++^ (Bagenal et al., 2015). We therefore adjusted the parameters of the model fluids to approximate this composition. We increased the charge of the magnetospheric plasma fluid from 1.0 to 1.5 e to account for the increased charge contributed by the S^++^. Since H^+^ is lighter and S^++^ is heavier relative to O^+^, we maintained the weight of the fluid to be 16 amu per ion, which effectively results in an ion fluid with a mass‐to‐charge ratio (M/Q) of ∼10.7 representing the magnetospheric plasma, consistent with previous in situ measurements (Bagenal et al., 2015). To calculate the photoionization, charge exchange, and recombination rates associated with the magnetospheric plasma we have retained the rates specified for O^+^ as given by Schunk and Nagy (2009).

For this parameter study we have improved the numerical grid from the previous version used in Harris et al. (2021). Since we decided to study atmospheres with small scale heights of 33 km, we added a layer of refinement such that the cells closest to Europa's surface are ∼7 km long in the radial direction, allowing for about 4 layers of cells inside the first 33 km of the atmosphere.

Most parameters of the simulations were held constant across the study. At the outer boundary of the simulation, we set the magnetospheric plasma velocity to 100 km/s, along the X‐EPhiO direction. To simplify the analysis of the simulation results and eliminate asymmetries associated with the magnetic environment, we set the Jovian magnetic field to *B*_*J*_ = −400 nT, anti‐parallel to the Z‐EPhiO direction. Since we did not include any X‐EPhiO or Y‐EPhiO components in the background magnetic field, we did not include Europa's induced field in the simulations. Thus, the trends observed between the different simulation results are all due to variations in the atmosphere and the self‐consistent generation of the ionosphere, without intrinsic asymmetries caused by the magnetic field orientation. We set the temperature of the magnetospheric plasma fluid to 129 eV. The properties of the electrons and the calculation of source and loss terms associated with ionization, recombination, and charge exchange, as well as other numerical details, are the same as described in Harris et al. (2021).

### 2.1 Specification of Varied Model Parameters

The parameter study consists of 18 simulations covering the variation of three parameters: the magnetospheric plasma density, and the atmospheric surface density and scale height.

First, we varied the magnetospheric plasma density such that nine simulations were conducted with a low density of 20 cm^−3^, and nine with 100 cm^−3^. Our choice of low magnetospheric plasma density corresponds to the density observed by the *Galileo* PLS during the E4 flyby (Paterson et al., 1999). Our choice of high plasma density is more consistent with the densities derived from the *Galileo* PWS observations over many flybys, and may be a more nominal case for the magnetospheric plasma density at Europa's orbit (Bagenal et al., 2015; Kurth et al., 2001).

We prescribe the density of Europa's atmosphere using the following functional form (Rubin et al., 2015):

$$n_{L}\left(\vec{r}\right)=n_{0}\;\exp\left(-\frac{\left|\vec{r}-{\vec{r}}_{\text{Eu}}\right|}{H_{0}}\right)+n_{1}\;\exp\left(-\frac{\left|\vec{r}-{\vec{r}}_{\text{Eu}}\right|}{H_{1}}\right)$$

(1) $$n_{T}\left(\vec{r}\right)=n_{L}\left(\vec{r}\right)ꞏ\left(1+Aꞏ\cos\;\alpha \right)$$

The 3D density of O_2_ is described by the functions *n*_L_ over the leading hemisphere (0–180° in longitude) and by *n*_T_ over the trailing hemisphere (180°–360°). On the leading hemisphere, the density is composed of two components: the primary component described by surface density *n*_0_ and scale height *H*_0_, and the secondary population described by *n*_1_ and *H*_1_. On the trailing hemisphere, this density is enhanced due to the expected increase in surface sputtering on the upstream side by magnetospheric particles. The coordinate α measures the angular separation between the position, $\vec{r}$, and the apex of the trailing hemisphere, ranging from 0 to 90°. The parameter A controls the enhancement such that at the apex of the trailing hemisphere, where *α* = 0, $n_{T}=n_{L}ꞏ\left(1+A\right)$. This enhancement is designed to model the increased density of the atmosphere over the trailing hemisphere, where more sputtering occurs and therefore more O_2_ is generated. We set *A* = 2, corresponding to a factor of 3 difference in density between the leading and trailing hemispheres in accordance with estimations of the asymmetry of the sputtered flux of O_2_ determined by Cassidy et al. (2013).

This model represents the primary population of thermalized O_2_ close to Europa's surface described by *n*_0_ and *H*_0_, and the secondary population of sputtered, non‐thermal O_2_ described by *n*_1_ and *H*_1_ (Cassidy et al., 2007; Teolis et al., 2017). In this work, we varied the first component of the atmosphere model (details are described below), but fixed the secondary population, setting the parameters *n*_1_ = 4 × 10^3^ cm^−3^ and *H*_1_ = 600 km. These parameters were selected to model the large scale height O_2_ population as shown in Figure 5 of Teolis et al. (2017).

**Figure 1:**
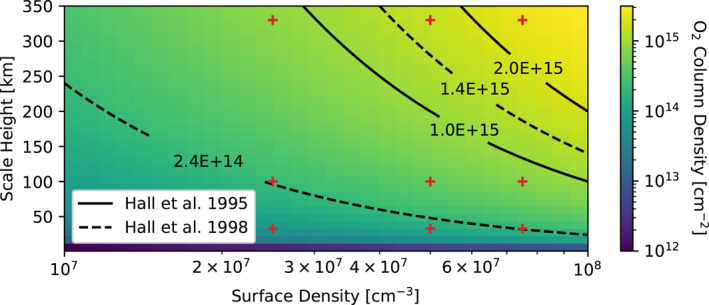
Probable O_2_ column densities for Europa's atmosphere. Black and white contours describe the upper and lower limits on the column density of Europa's atmosphere determined from observations of Europa's oxygen aurora by the Hubble Space Telescope (HST) (Hall et al., 1995, 1998). Red pluses mark the column density of the primary atmosphere population for each simulation in the parameter study. Due to the secondary population and the enhancement of the atmosphere density on the trailing hemisphere (Equation 1) the column density of the modeled atmosphere in each simulation varies over Europa's surface and on the trailing hemisphere is higher by up to a factor of 3 than shown here.

Within each of the two sets of simulations, we varied the primary component of Europa's atmosphere by setting the scale height (H_0_) to be either 33 km, 100 km, or 330 km. We then varied the surface density (*n*_0_) of the atmosphere to be either 2.5 × 10^7^, 5.0 × 10^7^, or 7.5 × 10^7^ cm^−3^. Figure 1 illustrates how these nine different atmosphere models span the range of Europa's column density determined by Hall et al. (1995, 1998), and Table 1 gives the average, minimum, and maximum column densities for each atmosphere model. The minimum column density occurs on Europa's leading hemisphere, the maximum at the apex of the trailing hemisphere, and the average is calculated over the whole surface. The observations used by Hall et al. (1995, 1998) to determine the range shown in Figure 1 were conducted in 1994 and 1996, and therefore if the structure of the atmosphere varied over this time period the range would encompass that behavior. Note that the two atmospheres in this study with the most extreme column densities (the lowest and the highest) fall outside the ranges established by Hall et al. (1995, 1998). The simulations that use these atmospheres represent edge cases and provide upper and lower bounds on the results.

**Table 1:** O_2_ Column Density Over Europa's Surface for Each Atmosphere

Scale height (km)	Low surface density, 2.5 × 10^7^ cm^−3^			Medium surface density, 5.0 × 10^7^ cm^−3^			High surface density, 7.5 × 10^7^ cm^−3^		
	Av. CD	Min. CD	Max. CD	Av. CD	Min. CD	Max. CD	Av. CD	Min. CD	Max. CD
	(10^14^ cm^−2^)			(10^14^ cm^−2^)			(10^14^ cm^−2^)		
330	11.6	8.25	24.8	23.2	16.5	49.5	34.7	24.8	74.3
100	3.51	2.50	7.51	7.02	5.00	15.0	10.5	7.50	22.5
33	1.16	0.83	2.48	2.32	1.65	4.96	3.48	2.48	7.43

*Note*. In each table entry, we give the average column density over Europa's whole surface followed by the minimum and maximum column densities. For each atmosphere, the maximum column density occurs at the apex of the trailing hemisphere, while the minimum occurs uniformly over the leading hemisphere. In the figures, we use the minimum column density value to order the different simulations.

## 3 Results

As described above, the parameter study is comprised of 18 steady‐state simulations, with nine simulations conducted with lower magnetospheric plasma density and the remaining nine conducted with high plasma density. Within each set of nine, the scale height and surface density of the O_2_ atmosphere were varied. Each simulation resulted in 3D solutions for the bulk plasma parameters and the local magnetic fields according to these different scenarios. Here, we summarize the simulation results and identify the general trends that emerge in these 18 simulations.

### 3.1 Ionosphere Density and Mass Loading

**Figure 2:**
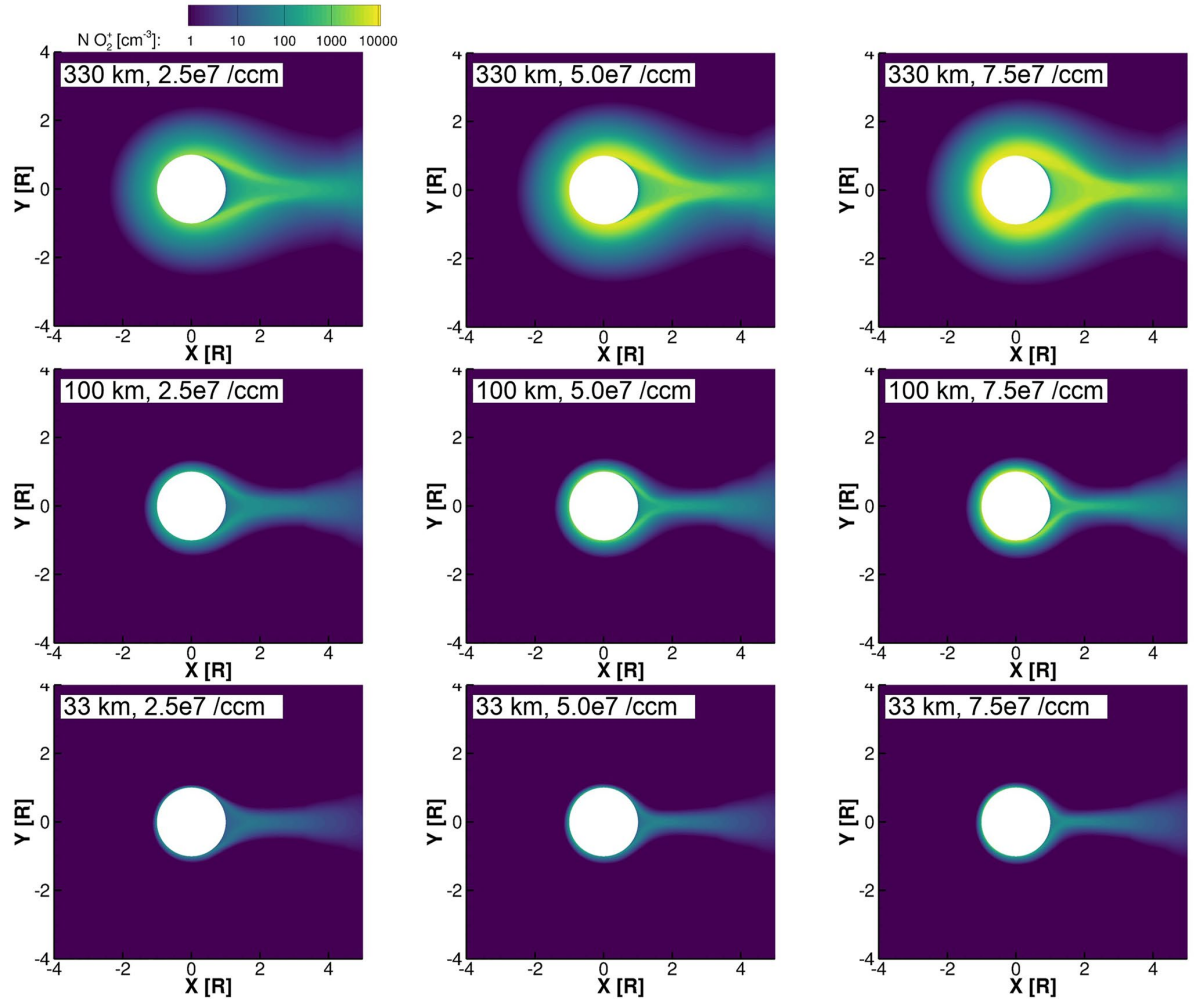
Density of O_2_^+^ in the equatorial plane for the simulations with low magnetospheric plasma density. The left column shows simulations with low surface density atmospheres (2.5 × 10^7^ cm^−3^), the center column shows simulations with medium surface density atmospheres (5.0 × 10^7^ cm^−3^), and the right column shows simulations with high surface density atmospheres (7.5 × 10^7^ cm^−3^). The top row shows simulations with large scale height atmospheres (330 km), the middle row shows simulations with medium scale height atmospheres (100 km), and the bottom row shows simulations with small scale height atmospheres (30 km).

**Figure 3:**
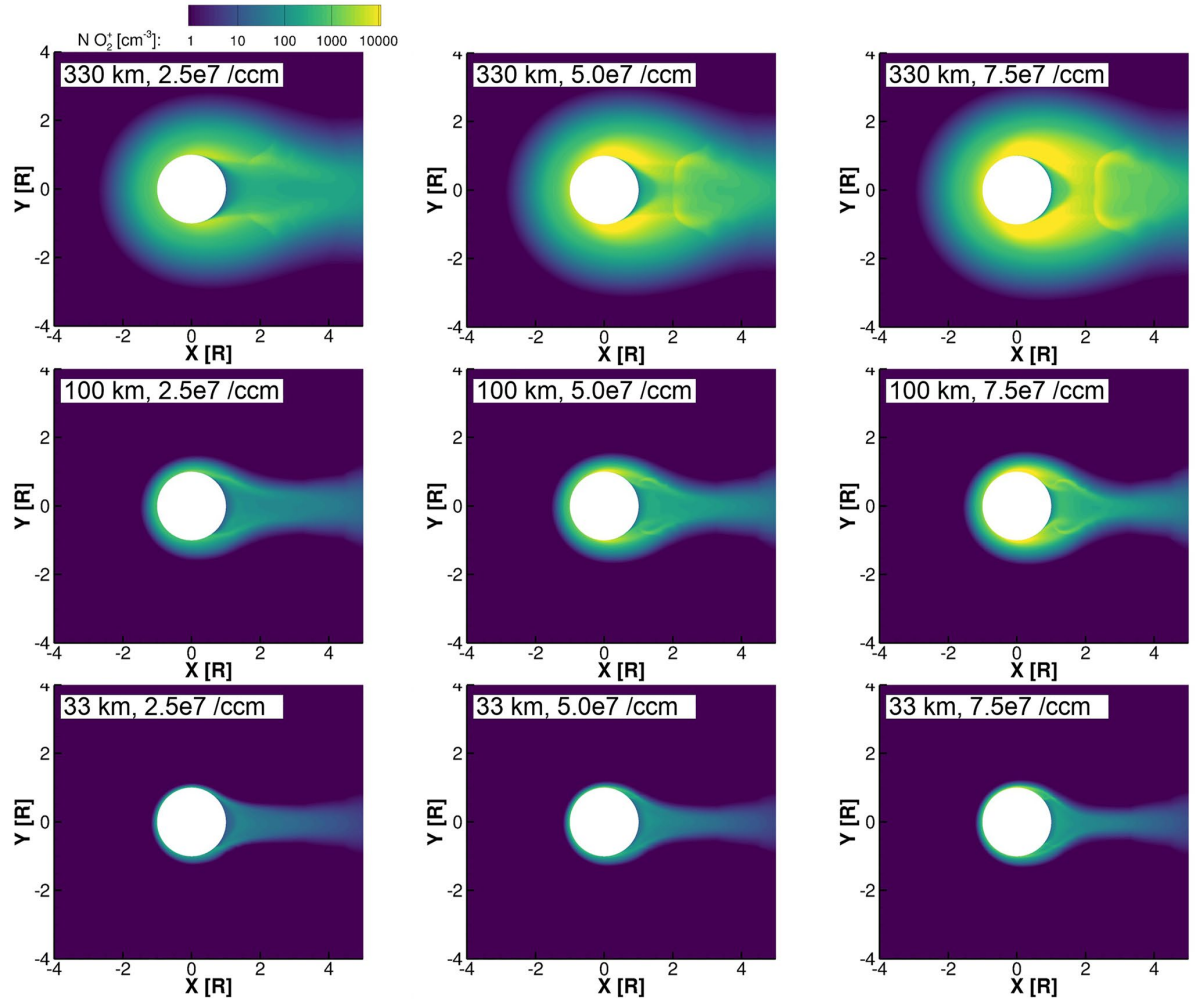
Density of O_2_^+^ in the equatorial plane for the simulations with high magnetospheric plasma density. The panels are ordered as in Figure 2.

Figures 2 and 3 show the density of O_2_^+^ in the equatorial plane for the simulations with low and high magnetospheric plasma density, respectively. In the simulations, O_2_^+^ is the primary component of Europa's ionosphere, and therefore, its density contours illustrate the boundaries and features of the resulting ionosphere. In general, we see that the simulations with high magnetospheric plasma density (Figure 3) developed denser ionospheres; this is consistent with the results of Harris et al. (2021), where we found that the column density of the ionosphere on the trailing hemisphere increased approximately linearly with the magnetospheric plasma density. In the top rows of both Figures 2 and 3, we see that in the simulations with the largest scale‐height atmospheres the region influenced by Europa's ionosphere extends far from Europa's surface and the plasma wake is loaded with high densities of ions. Where the atmosphere scale height is small, the ionosphere is confined close to Europa's surface (bottom rows of Figures 2 and 3).

**Figure 4:**
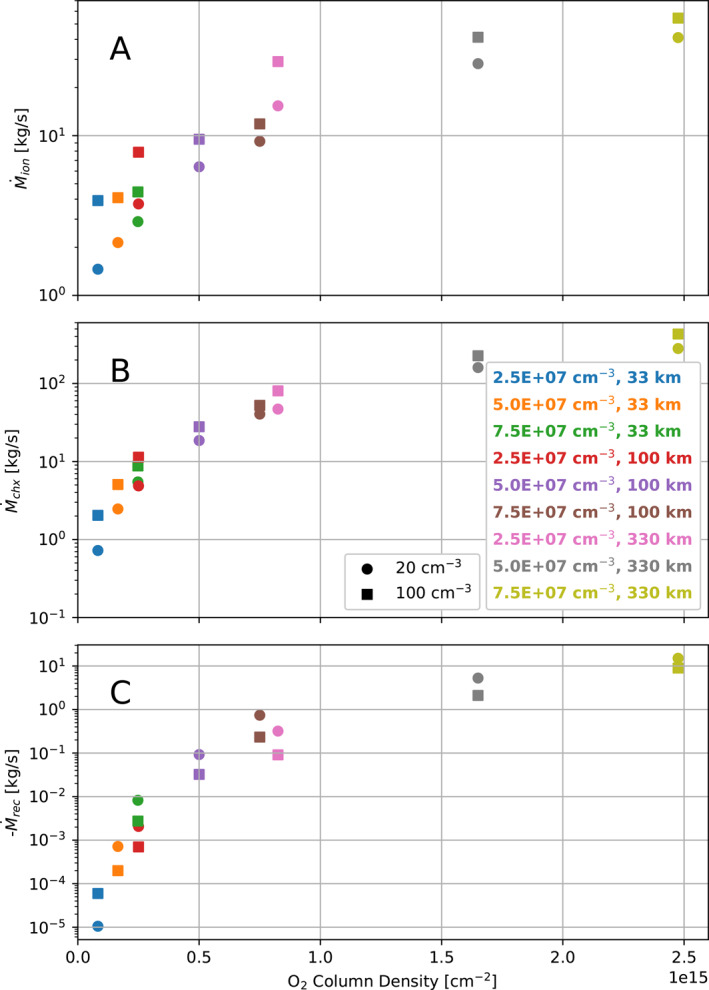
Mass production due to ionization (a) and charge exchange (b), and mass lost due to dissociative recombination (c) in each simulation. Circle markers indicate simulations with low magnetospheric plasma density (20 cm^−3^), while squares indicate high magnetospheric plasma density (100 cm^−3^). Markers are ordered on the *X*‐axis according to the minimum column density for the atmosphere in the simulation (see Table 2).

Table 2 gives the total mass production and loss rates for the simulation domain inside 5 R_Eu_, summed over all the ion fluids, for each simulation. Figure 4 presents these numbers ordered according to the minimum column density of the atmosphere in each simulation. We show the production rate of cold ions by photoionization and electron impact ionization, the rate at which mass undergoes charge exchange, and the rate at which mass is lost from the ion fluids to dissociative recombination. The primary contributions to each rate come from the O_2_^+^ fluid. In the simulations with lower atmosphere surface densities and smaller scale heights, ionization is the most significant of these processes. However, as the atmosphere density and scale height increases, charge exchange becomes more significant. Table 2 and Figure 4 list only the effects on mass, but each of these processes also affect the momentum and pressure of the ion fluids (Rubin et al., 2015).

**Table 2:** Mass Production and Loss Rates in Each Simulation

Process	*N*_Mag_ (cm^−3^)	Scale height (km)	Low surface density, 2.5 × 10^7^ cm^−3^ (kg/s)	Medium surface density, 5.0 × 10^7^ cm^−3^ (kg/s)	High surface density, 7.5 × 10^7^ cm^−3^ (kg/s)
Ionization^a^	20	330	15.4	28.2	41.1
		100	3.74	6.38	9.22
		33	1.46	2.14	2.90
	100	330	29.1	41.3	54.4
		100	7.88	9.49	11.8
		33	3.92	4.09	4.44
Charge exchange^b^	20	330	47.0	160	280
		100	4.87	18.6	40.5
		33	0.72	2.46	5.47
	100	330	80.3	226	430
		100	11.4	27.9	52.4
		33	2.04	5.07	8.76
Recombination^c^	20	330	0.320	5.28	15.0
		100	2.07e−3	9.24e−2	0.741
		33	1.05e−5	7.16e−4	8.23e−3
	100	330	9.10e−2	2.11	8.96
		100	7.00e−4	3.24e−2	0.233
		33	5.95e−5	1.99e−4	2.74e−3

*Note.* Mass is summed over the ion fluids. For all processes the primary contributor is the O_2_^+^ fluid.

^a^ Mass produced by photoionization and electron impact ionization.

^b^ Mass undergoing charge exchange with neutral O_2_.

^c^ Mass lost to dissociative recombination.

These rates for charge exchange are consistent with previous multi‐fluid MHD models when simulations with similar atmospheres are compared. In their simulations, Jia et al. (2018) found a charge exchange rate of ∼27 kg/s. They used an atmosphere with surface density of 4 × 10^7^ cm^−3^ and scale height of 100 km in the primary component, and upstream plasma density of 500 cm^−3^. Their simulations are therefore most comparable to our simulation with atmosphere surface density of 5 × 10^7^ cm^−3^, scale height of 100 km/s, and upstream plasma density of 100 cm^−3^, where the charge exchange rate is 27.9 kg/s. Harris et al. (2021) included simulations with variable upstream plasma densities in their study, but in all cases the atmosphere was specified with surface density of 2.5 × 10^7^ cm^−3^ and scale height of 100 km. The charge exchange rate was 5.13 kg/s in simulations with upstream plasma density of 20 cm^−3^ and 11.45 kg/s in a simulation with upstream plasma density of 130 cm^−3^. In this study, our two simulations with a similar atmosphere exhibited charge exchange rates of 4.87 (20 cm^−3^ upstream plasma density) and 11.4 kg/s (100 cm^−3^ upstream plasma density). There are no comparable simulations published for our most extreme simulations with very dense, extended atmospheres.

In Figure 4, we note that as the column density of the atmosphere increases the rate of mass added to the ion fluids (through ionization and charge exchange) begins to level off, as does the rate of mass lost to recombination. The ionization and charge exchange mass loading rates increase with the density of the atmosphere, and the recombination rate increases with the density of the ion fluids. They are all limited by the electron temperature. These rates increase when the O_2_ column density of the atmosphere is increased because more O_2_ is available to be ionized or to engage in charge exchange, and more ionospheric plasma is generated and available to undergo dissociative recombination. However, these interactions all tend to decrease the temperature of the electrons, reducing the energy available to support them, and therefore their growth as mass sources or losses is limited.

### 3.2 Magnetic Fields

**Figure 5:**
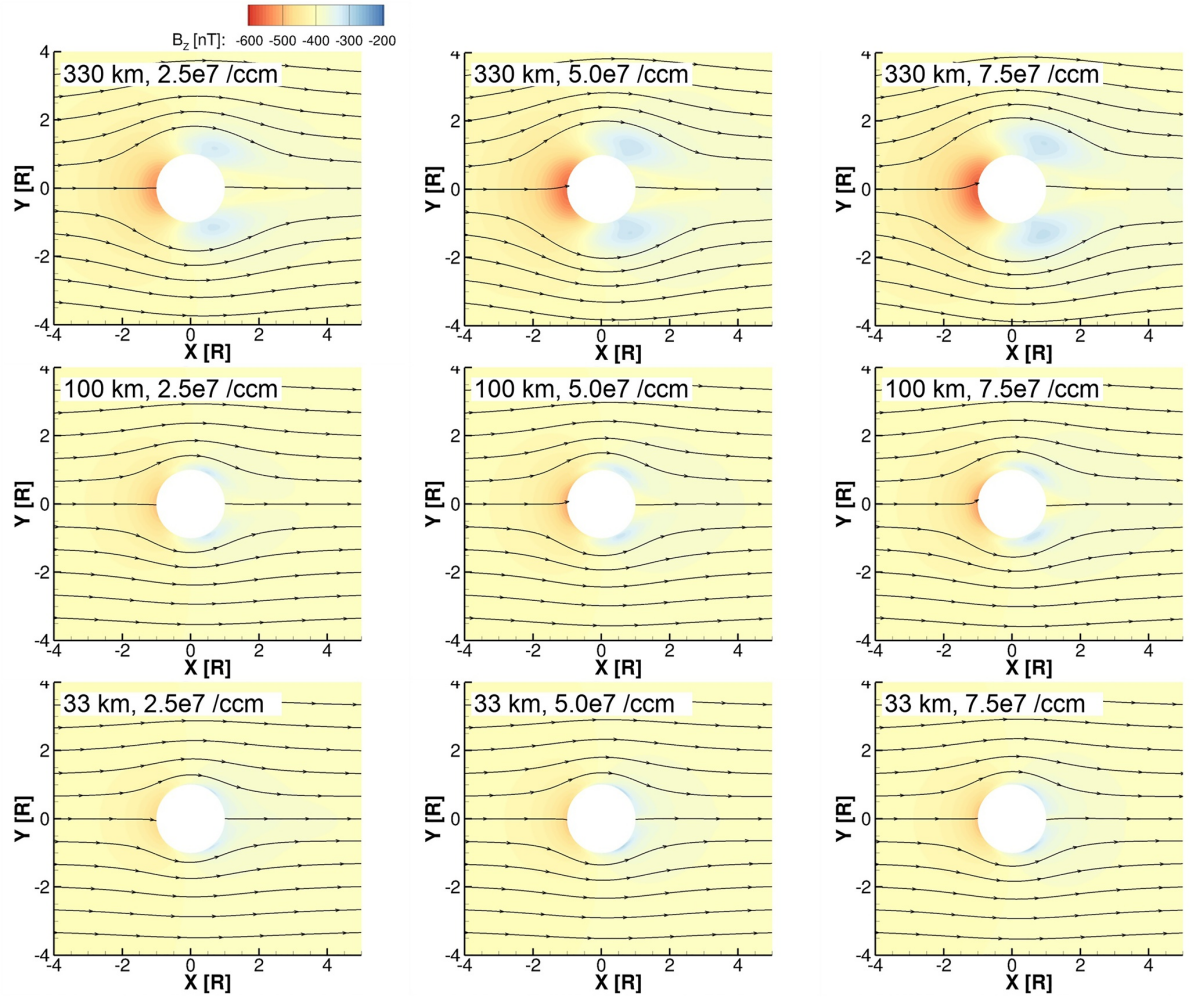
Color contours show the magnetic field in the equatorial plane for the simulations with low magnetospheric plasma density. Streamlines indicate the direction of the charge‐averaged velocity ${\vec{U}}_{q}$ in the XY‐EPhiO plane. The panels are ordered as in Figure 2.

**Figure 6:**
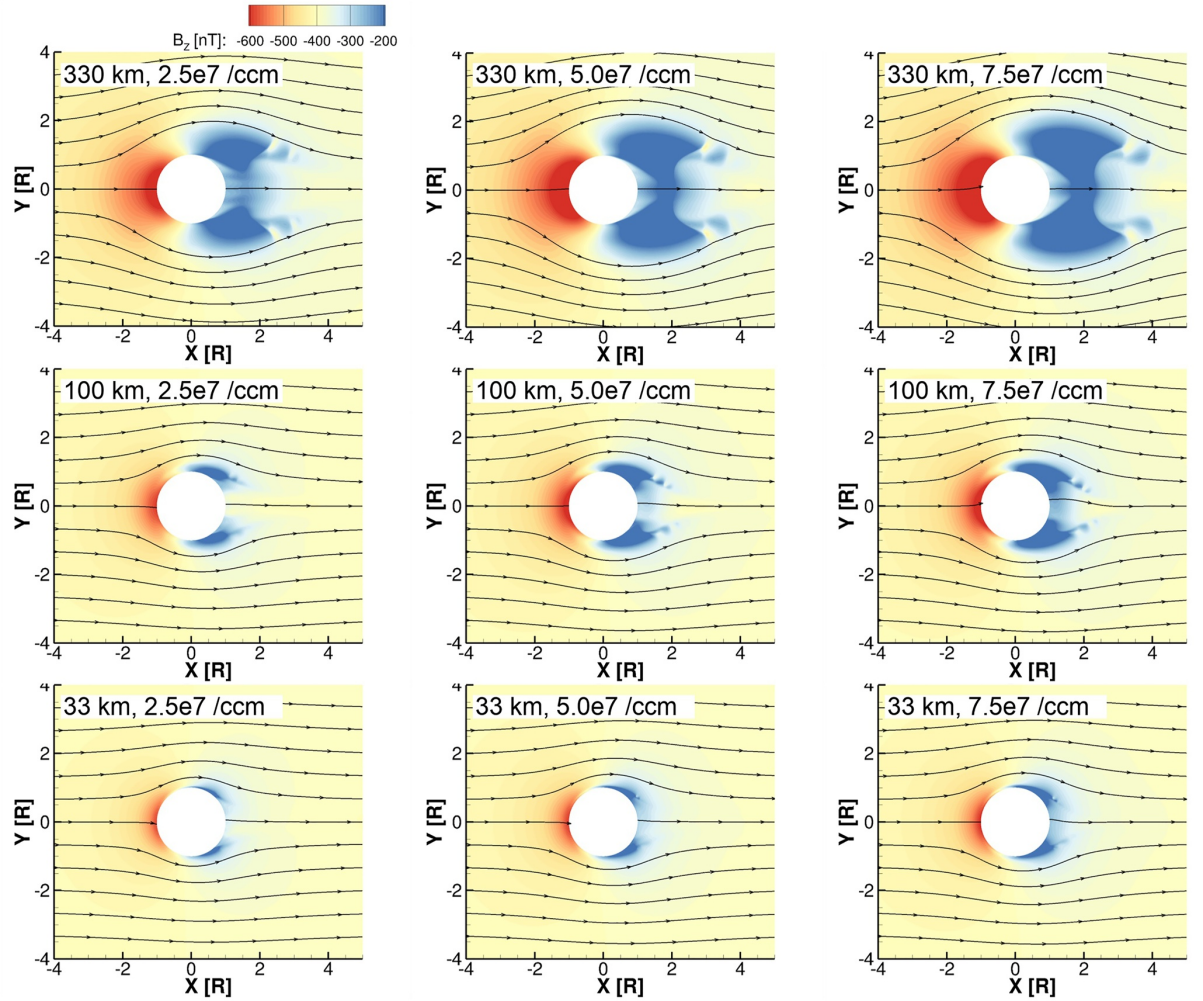
Color contours show the magnetic field in the equatorial plane for the simulations with high magnetospheric plasma density. Streamlines indicate the direction of the charge‐averaged velocity ${\vec{U}}_{q}$ in the XY‐EPhiO plane. The panels are ordered as in Figure 2.

Figures 5 and 6 show the Z‐EPhiO component of the magnetic field (*B*_*Z*_) and streamlines of the charge‐averaged velocity (${\vec{U}}_{q}$) in the equatorial plane. The simulated perturbations are roughly symmetric around the X‐EPhiO axis as a result of our choice of symmetric Jovian background field as input for all simulations presented here. For these simulations, the strength of perturbations to *B*_*Z*_ indicates that the magnetic field is compressed, or piled‐up, on the upstream side of the interaction as the flow of magnetospheric magnetic field, which is frozen‐in to the magnetospheric plasma, is forced to slow due to the interaction of the magnetospheric plasma with Europa's ionosphere. In Figure 5, which shows the simulations with low magnetospheric plasma density, the upstream magnetic field pile‐up (red) is weaker than in Figure 6, which shows the simulations with high plasma density and consequently higher ionosphere densities. In both figures, we observe that the spatial extent of the upstream magnetic field pile‐up, as well as the distance from Europa's surface at which streamlines start diverting from their ambient, straight paths, increases with the scale height of the atmosphere due to the increased extent of the ionosphere.

**Figure 7:**
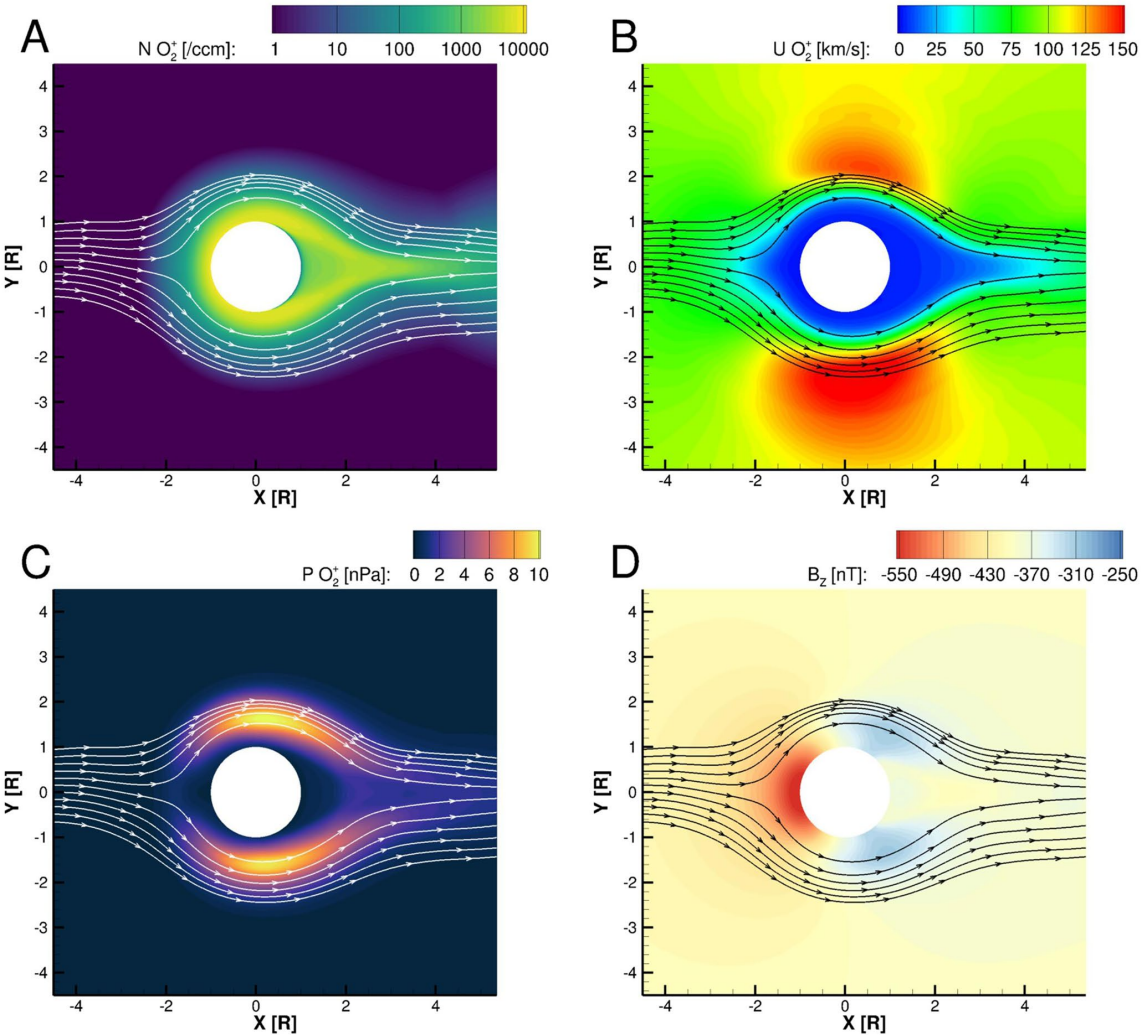
Bulk plasma properties of O_2_^+^ and *B*_*Z*_ in the equatorial plane for the low magnetospheric plasma density, high atmosphere surface density, and high atmosphere scale height simulation (top right panel in Figures 2 and 5). Panels (a–c) show, respectively, the number density, speed, and pressure of the O_2_^+^ magnetohydrodynamic fluid, while Panel (d) shows *B*_*Z*_. Streamlines in all four panels indicate the velocity of O_2_^+^ in the equatorial plane.

In both Figures 5 and 6, we observe that in each simulation on the downstream side of the interaction, where the magnetic field is relatively depressed (blue), there are two local minima of the *B*_*Z*_ magnetic field, one on the sub‐Jovian (+Y) flank and one on the anti‐Jovian (−Y) flank. By comparing Figure 2 with Figure 5 and Figure 3 with Figure 6, we observe that the regions of depressed magnetic field correspond approximately to the locations where the ionospheric plasma density is highest in each simulation. Figure 7c shows the pressure of the O_2_^+^ fluid in the simulation corresponding to the upper rightmost panel of Figure 5. Two factors act to increase the pressure on Europa's flanks. Close to Europa's surface where the atmospheric neutral density is high, relatively large plasma pressure arises from dense ionospheric plasma produced through ionization of neutrals. Farther from the surface, the thermal plasma pressure increases as newly generated ions (O_2_^+^ and O^+^), either through ionization or charge‐exchange, are picked up by increased flow speeds on the two flanks (Figure 7b). To maintain pressure balance, the magnetic pressure $B^{2}/2\mu_{0}$ decreases, resulting in the magnetic field depressions.

In the high magnetospheric plasma density simulations with atmospheres of 330 km scale height (top rows of Figures 3 and 6), the plasma wake is much more extended and densely populated with O_2_^+^ ions, and there are gradients in density and magnetic field. These features can be understood by considering the flux tubes that flow downstream, moving with the magnetic field and plasma through the interaction as indicated by the streamlines in Figure 6. In a crescent‐shaped region close to Europa's surface on the downstream side, the plasma density is depleted because the flux tubes that reach this area must flow directly over Europa's surface, where plasma is absorbed. Farther downstream, the streamlines in Figure 6 show that these flux tubes have been diverted and traveled through the flanks of the interaction before reconvening toward the *X*‐axis. As these flux tubes travel through the interaction they continue to ionize neutral O_2_ and produce plasma. Eventually, along this path, the electron energy in the flux tube is depleted, and plasma production decreases. In general, along a line of constant *X* through the plasma wake the plasma density increases to maxima within the flanks and dips where *Y* = 0 due to this effect. These gradients in the density cause corresponding gradients in the magnetic field of the plasma wake. In these three simulations, the scale height of the atmosphere is the largest, and the effects of flow diversion are relatively strong, causing these effects to appear stronger at farther extent from the moon compared to the other simulations.

### 3.3 Electron Density

**Figure 8:**
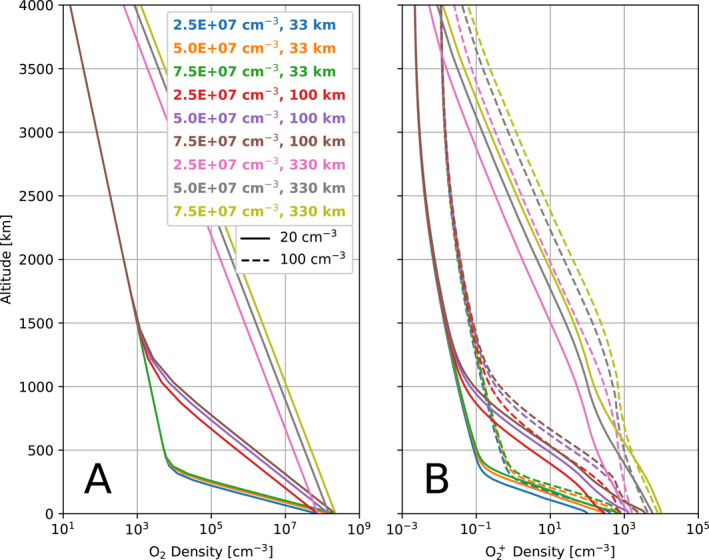
Comparison of atmosphere and ionosphere density profiles along the upstream/‐X‐EPhiO axis. Panel (a) shows the O_2_ density for each atmosphere in the study. Panel (b) shows the corresponding density of O_2_^+^. In Panel (b), solid lines give the density from the simulations with low magnetospheric plasma density, while the dashed lines correspond to simulations with high magnetospheric plasma density.

To better compare the densities of plasma between simulations, in Figure 8 we show altitude profiles of the density of the atmospheres and ionospheres for every simulation in the study. Figure 8a presents the prescribed density of the atmospheres along the ‐*X*‐EPhiO axis. The density decreases monotonically with distance from the surface. After a radial distance of several atmosphere scale heights, the rate of decrease in density drops as the primary component of the atmosphere becomes less significant than the secondary population which has a low density but a larger scale height. For the highest scale height simulations, this change in slope occurs at higher altitudes than shown here. In Figure 8b, we show the corresponding density of the ionosphere in each simulation. Near Europa's surface the density of O_2_^+^ is primarily controlled by the ion production rate, which is proportional to the neutral density of the atmosphere. Therefore, the rate at which the density of O_2_^+^ decreases changes at the same altitudes where the changes occurred in the atmospheric density, at about 400 km for the 33 km scale heights, about 1,250 km for the 100 km scale heights, and at farther distances for the 330 km scale heights.

Figure 8b also illustrates that the density of the ionosphere generally increases with higher magnetospheric plasma density (dashed lines), consistent with the findings of Harris et al. (2021). This occurs because the increased magnetospheric plasma density increases the electron density, and therefore, colder O^+^ and O_2_^+^ are produced by electron impact ionization. However, there are four atmosphere cases where the ionosphere density at low altitudes for the low magnetospheric plasma density simulation slightly exceeds that for the high plasma density simulation. This occurs for the four atmospheres with medium or high scale height (330 km or 100 km) and medium or high surface density (5.0 × 10^7^ cm^−3^ or 7.5 × 10^7^ cm^−3^). In these simulations, the ionosphere extends farther from the surface of the moon and more efficiently shields the surface from the magnetospheric plasma. Therefore, the effects of the increased magnetospheric plasma density are reduced at low altitudes, and the ionosphere densities are similar between each pair of simulations with the same atmosphere.

**Figure 9:**
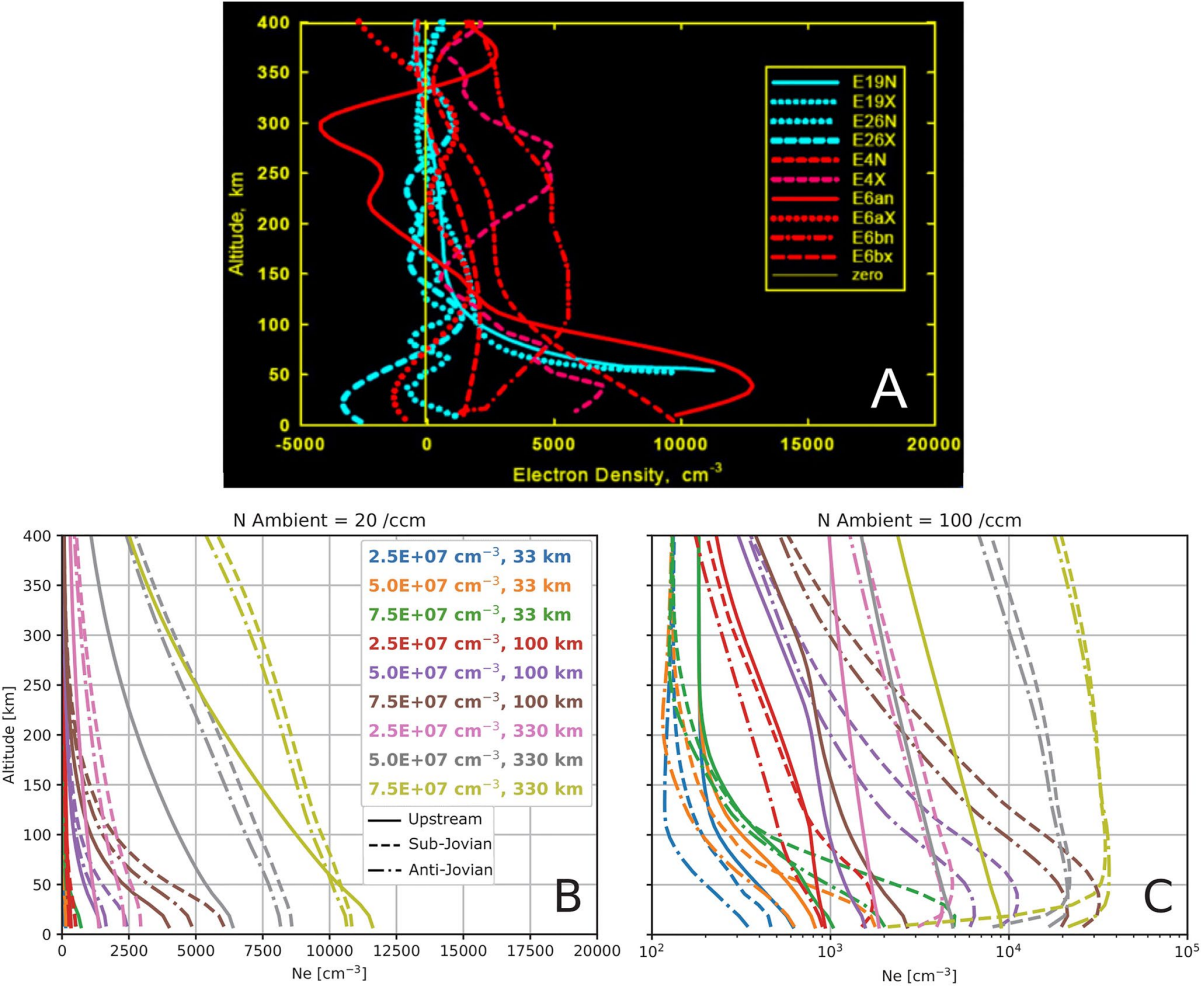
Electron density profiles at low altitudes. Panel (a) shows data measured by *Galileo* radio occultations, reproduced from McGrath et al. (2009), Figure 7. In Panels (b and c) solid lines denote the simulated electron density along the upstream/‐X‐EPhiO axis, dashed lines correspond to the sub‐Jovian/+Y‐EPhiO axis, and dash‐dot lines correspond to the anti‐Jovian/‐Y‐EPhiO axis. Panel (b) gives the electron density for the simulations with low magnetospheric plasma density, while Panel (c) shows the simulations with high magnetospheric plasma density. Note that the *X* axes differ between Panel (b) and Panel (c) For Panel (b), the range was chosen for easy comparison with the *Galileo* radio occultation measurements shown in McGrath et al. (2009), Figure 7. For Panel (c), the range was increased and the *X*‐axis scaled logarithmically to show the maximum electron density and variations at lower altitudes.

In Figure 9, we examine the variability of the electron density focusing on the region within 400 km of Europa's surface. For each simulation, we show the electron density along the upstream direction (‐X‐EPhiO) as well as on the sub‐ and anti‐Jovian flanks (+Y and ‐Y‐EPhiO). Figure 9b shows the electron density for the simulations with low magnetospheric plasma density. In these cases, the electron density is generally consistent with the densities derived from the *Galileo* radio occultation experiment shown in Figure 9a (McGrath et al., 2009). These electron density profiles were observed at a variety of locations over Europa's surface, just as we sample different locations for the profiles shown in Figures 9b and 9c. Figure 9c shows the electron density for the simulations with high magnetospheric plasma density. While the highest density ionospheres are significantly denser than the *Galileo* electron densities (which do not exceed 15,000 cm^−3^), most of the high magnetospheric plasma density simulations still produced ionospheres similar to the *Galileo* occultation profiles. Thus, we find that reasonable variations in Europa's atmosphere can cause the electron density to vary by multiple orders of magnitude at the same altitudes, in agreement with the observations.

Figure 9 demonstrates the significant variability of Europa's ionosphere not only with variation of the atmosphere, but in different regions within the plasma interaction. In both plots different line styles indicate simulation output extracted along the upstream/‐*X*‐axis (solid), sub‐Jovian/+*Y*‐axis (dash‐dash), and anti‐Jovian/‐*Y*‐axis (dash‐dot). Figure 9c shows that in the simulations with low magnetospheric plasma density, the electron density is generally higher on the sub‐Jovian side (dashed) of the plasma interaction than the anti‐Jovian (dash‐dot). This asymmetry is associated with the multi‐fluid properties of the plasma interaction. The electron number density is calculated as the sum of the ion number densities, and is dominated by the number density of the ionospheric fluids, which greatly exceed that of the magnetospheric ion fluid (Harris et al., 2021). Therefore, this asymmetry in the electron density is caused by the enhancement of the ionospheric plasma densities on the sub‐Jovian side of the moon. This density enhancement occurs because the flow speed of the ionospheric plasma fluids is lower on the sub‐Jovian side of the interaction (Figure 7b), permitting plasma to preferentially accumulate on the sub‐Jovian flank. This asymmetry in the velocity arises due to Lorenz force effects caused by the differential motion of the ion fluids with respect to the magnetic field, and is discussed by Harris et al. (2021).

### 3.4 Ionosphere Column Density

**Figure 10:**
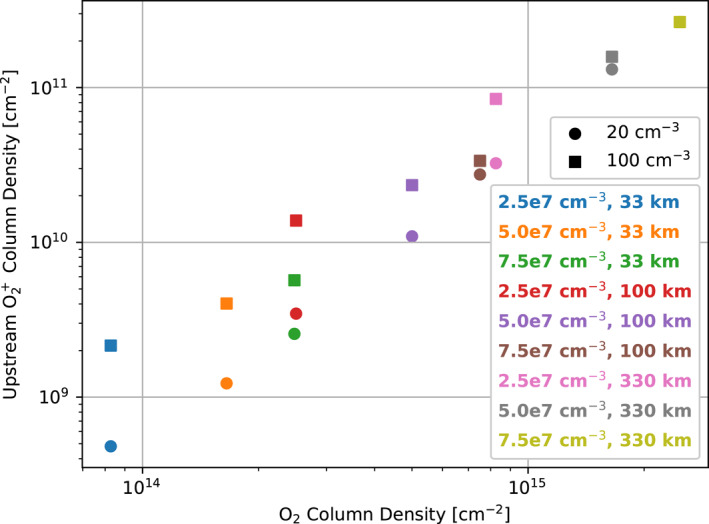
Column density of O_2_^+^ integrated along the upstream/‐X‐EphiO axis. Circle markers indicate simulations with low magnetospheric plasma density (20 cm^−3^), while squares indicate high magnetospheric plasma density (100 cm^−3^). Markers are ordered on the *X*‐axis according to the minimum column density for the atmosphere in the simulation (see Table 2). Note that the markers for the simulations with the highest atmosphere column density atmosphere overlap each other.

Figure 10 compares the column density of Europa's ionosphere integrated along the upstream/‐*X*‐EPhiO axis with the minimum column density of the atmosphere, essentially integrating the curves shown in Figure 8b. The column density of the ionosphere increases with that of the atmosphere in a ratio of 1:10^4^. In other words, to increase the column density of the ionosphere by a certain amount requires that the column density of the atmosphere should increase by 10,000 times that amount. In Harris et al. (2021), the upstream column density of O_2_^+^ ranged from 2.5 × 10^9^ to 3.4 × 10^10^ cm^−2^. Here, the O_2_^+^ column density ranges from 4.8 × 10^8^ to 2.7 × 10^11^ cm^−2^ for the simulations with low magnetospheric plasma density and from 2.2 × 10^9^ to 2.7 × 10^11^ cm^−2^ for those with high plasma density. In this study, we therefore observe a larger range of variation in the ionosphere column density that encompasses the regime explored by Harris et al. (2021).

In Figure 10, we also observe that as the atmosphere column density increases, the ionosphere column density converges to the same values for the simulations with the same atmospheres. This occurs due to the shielding effect observed in Figure 8b. For simulations with low atmosphere column densities, the ionosphere column density is always increased in the simulation with high magnetospheric plasma density (Harris et al., 2021). As the atmosphere column density increases, the resultant ionosphere becomes denser and more extended, and therefore shields the surface more completely from the magnetospheric plasma such that at low altitudes the ionosphere density is mainly determined by the density of the atmosphere. Thus, simulations with the same atmosphere result in similar ionosphere densities at low altitudes. Since most of the ionosphere column density is contributed at low altitudes where the ionosphere density is highest, this results in the same ionosphere column density for simulations with the same atmosphere parameters.

## 4 Precipitation of Magnetospheric Plasma

We have described the general trends in variation of the ionospheric structure and the resulting magnetic field perturbations illustrated by the results of this parameter study. These properties of the plasma interaction control the intensity and spatial patterns of the precipitation of magnetospheric plasma onto Europa's surface. This precipitation in turn contributes to the sputtering process that generates Europa's atmosphere. To better understand the coupling between the plasma environment and Europa's atmosphere, we now analyze the simulations of this study to characterize the effects of Europa's atmosphere on the precipitation of magnetospheric plasma.

**Figure 11:**
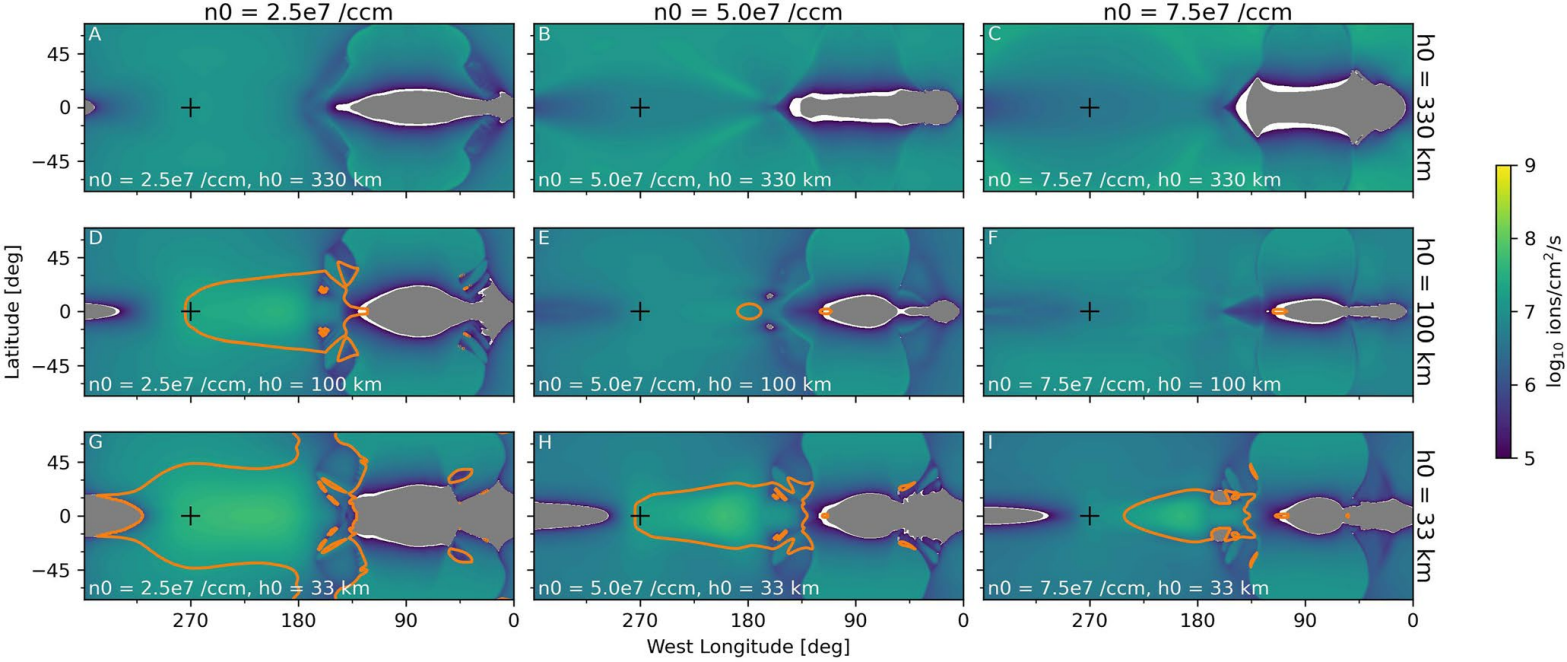
Maps of downward flux of the magnetospheric plasma fluid in each of the simulations with low upstream plasma density (20 cm^−3^). The panels are ordered as in Figure 2. Gray regions block out locations where the net flux of plasma is upward, flowing away from the surface. Black pluses mark the apex of the trailing hemisphere, at 0° latitude and 270°W longitude. Orange contours describe regions where the temperature of the precipitating plasma exceeds 100 eV.

**Figure 12:**
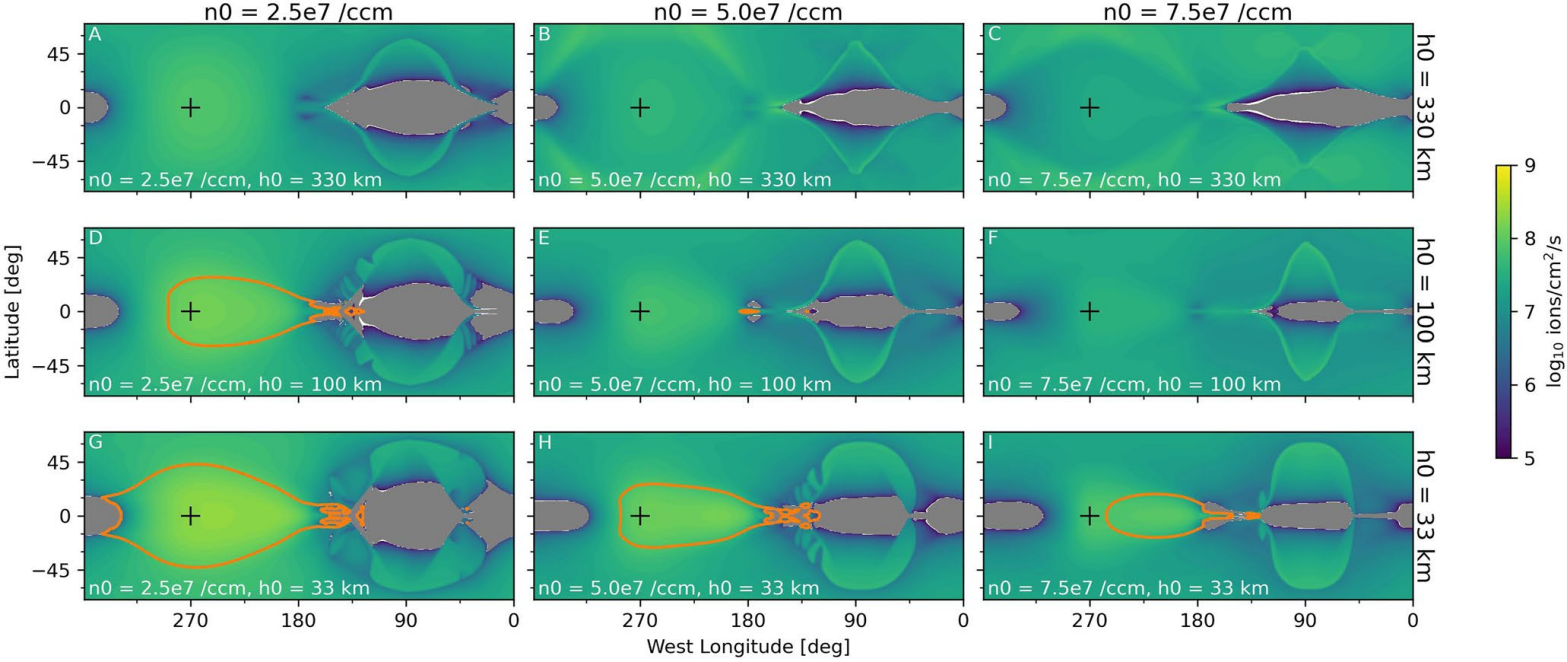
Maps of downward flux of the magnetospheric plasma fluid in each of the simulations with high upstream plasma density (100 cm^−3^). The panels are ordered as in Figure 2. Annotations are as described for Figure 11.

Figures 11 and 12 show maps of the downward flux of magnetospheric plasma from each simulation in the study. Orange contours mark where the temperature of the precipitating plasma exceeds 100 eV, which we have identified because above this temperature the thermal plasma may make an appreciable contribution to sputtering (see, e.g., the sputtering study results compiled in Figure 1 of Vorburger and Wurz (2018)). The intensity of the precipitating flux is uniformly increased in the simulations with high magnetospheric plasma density (Figure 12). Within each figure, trends in the temperature and precipitation patterns emerge with the varying surface density and scale height of the atmosphere. For atmospheres with smaller scale heights (toward the bottom of each figure), or lower surface densities (toward the left in each figure), more magnetospheric plasma with temperatures higher than 100 eV precipitates. As was observed by Harris et al. (2021), on the leading hemisphere (0°–180°W longitude) in all simulations we observe a patch of no precipitation near the equator, and low precipitation from middle to high latitudes (∼3 × 10^6^ cm^−2^ s^−1^ in the simulations with low magnetospheric plasma density, ∼3 × 10^7^ cm^−2^ s^−1^ for those with high magnetospheric plasma density). Addison et al. (2021) also observed precipitation on Europa's leading hemisphere in their analysis of thermal plasma precipitation in hybrid plasma simulations.

On the trailing hemisphere (180°–360°W longitude) many of our simulation results differ from Harris et al. (2021). In Harris et al. (2021), which used an atmosphere with surface density of 2.5 × 10^7^ cm^−3^ and scale height of 100 km, for all cases the precipitation rate reached a maximum near the apex of the trailing hemisphere (0° latitude and 270° W longitude, indicated by black plus symbols) and decreased with angular distance from this point. In this study, we find that for the cases with the largest atmosphere scale heights the maximum intensity of precipitating plasma does not occur near the apex of the trailing hemisphere. In particular, in Figures 11b, 11c, 12b, and 12c we observe a lens‐like pattern of decreased flux around the apex of the trailing hemisphere and higher flux in a rim around the edge of the trailing hemisphere. This contrasts with, for example, Figure 12i, in which the precipitation peaks near the apex of the trailing hemisphere.

The streamlines and plasma properties shown in Figure 7 come from the same simulation shown in Figure 11c and the top right panels of Figures 2 and 5. We see in Figure 7 that due to the high density and large scale height of the atmosphere in this simulation, Europa's ionosphere extends about 0.5 R_Eu_ away from the surface of the moon on the upstream side of the interaction. The top right panel of Figure 2 shows that this simulation produced the densest ionosphere of all the simulations with low magnetospheric plasma density. We also note that the top right panel of Figure 5 shows that, due to this dense, extended ionosphere, this simulation produced the strongest pile‐up of magnetic field on the trailing hemisphere of these nine simulations. This pile‐up of magnetic field shields the surface on the trailing hemisphere, pushing plasma that would have precipitated near the apex of the trailing hemisphere away from the *X*‐EPhiO axis radially, so that it precipitates into a ring‐like shape.

In Figures 11 and 12, we also observe that the diversion of plasma is asymmetric such that plasma is more strongly excluded from Europa's surface on the sub‐Jovian side of the moon, whereas plasma is able to more easily reach the surface on the anti‐Jovian side. This corresponds to the patches over the equator where no plasma precipitates on the sub‐Jovian side (near 360°, as seen in Figures 11a, 11d and  , and in all panels in Figure 12) and where relatively warm plasma precipitates on the anti‐Jovian side (near 180°, as seen particularly strongly in Figures 11g–11i and in Figures 12g–12i). This occurs because the ionosphere tends to be denser on the sub‐Jovian side than on the anti‐Jovian side (Figure 9), and the magnetospheric plasma is therefore less able to penetrate the ionosphere on the sub‐Jovian side. The cause of this asymmetry is the Lorenz force acting on the ionospheric plasma, as discussed previously in Section 3.

**Figure 13:**
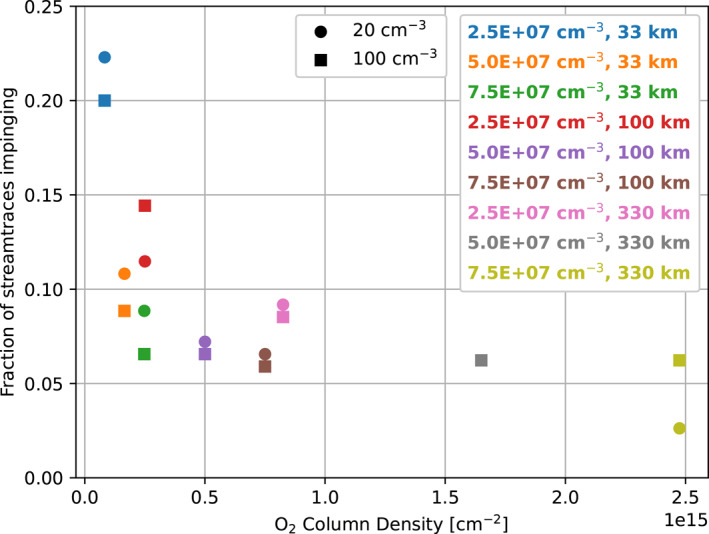
Fraction of plasma flow streamlines originating in the upstream cross‐section of Europa that impinge on Europa's surface in each simulation. Circle markers indicate simulations with low magnetospheric plasma density (20 cm^−3^), while squares indicate high magnetospheric plasma density (100 cm^−3^). Markers are ordered on the *X*‐axis according to the minimum column density for the atmosphere in the simulation (see Table 2). Note that the circle and square markers overlap each other for the simulations with the atmosphere with 5 × 10^7^ cm^−3^ surface density and 330 km scale height.

To better understand how plasma is diverted in the simulations, and to compare these results with those of Harris et al. (2021), we looked at the flow of the magnetospheric plasma. Figure 13 shows the fraction of plasma flow streamlines originating from the upstream that impinged on Europa's surface in each simulation. We seeded streamlines of the magnetospheric plasma flow upstream of Europa within the moon's cross‐section, then measured the number of streamlines that were diverted away from Europa's surface to calculate this fraction. Whereas in Harris et al. (2021), the authors found that 86%–90% of streamlines were diverted, with a variation of just 4% across the whole study, here we find much more variation. Across this study, the diversion ranged from 78% to 97%, or as shown in Figure 13, the percent of streamlines that reached Europa's surface ranged from 3% to 22%. As in Harris et al. (2021), we found that the magnetospheric plasma density made little difference in varying the fraction of diverted streamlines. There was less than 5% change between simulations with the same atmosphere parameters but different magnetospheric plasma densities, as indicated by the similar values between circle and square markers of the same color in Figure 13. The strongest effect on the streamlines is caused by increasing the surface density of the atmosphere; considering just the simulations with low magnetospheric plasma density and 33 km scale height atmospheres, we find that the percentage of diverted streamlines increases from 78% (surface density = 2.5 × 10^7^ cm^−3^) to 89% (surface density = 5.0 × 10^7^ cm^−3^) to 91% (surface density = 2.5 × 10^7^ cm^−3^). Considering impinging streamlines, the percentage decreases from 22% to 11%–9% for the same simulations.

This result agrees with the results of Saur et al. (1998), who developed a fluid model for Europa's plasma interaction to study the coupling between the plasma and the neutral atmosphere in uniform magnetic fields. The authors varied the surface density of the atmosphere, and therefore the column density of the atmosphere, and assessed the resulting system for mass balance between the different sources and losses in the model. Figure 3 of Saur et al. (1998) shows that they found that as the column density increased from 0.1 to 1.5 × 10^15^ cm^−2^ the effective radius of Europa as an obstacle to the plasma flow decreased significantly. Our findings are consistent with this result, as shown by Figure 13, which shows a sharp decrease in the percent of streamlines that impinge on Europa's surface through the same parameter space in atmospheric column density as that studied by Saur et al. (1998).

**Figure 14:**
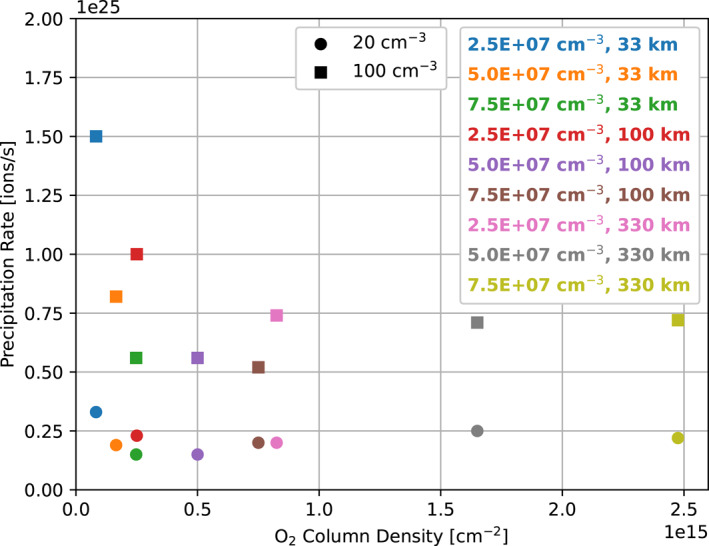
Integrated downward flux of magnetospheric plasma for each simulation. Circle markers indicate simulations with low magnetospheric plasma density (20 cm^−3^), while squares indicate simulations with high magnetospheric plasma density (100 cm^−3^). Markers are ordered on the *X*‐axis according to the minimum column density for the atmosphere in the simulation (see Table 2).

We integrated the downward number flux of the magnetospheric plasma over Europa's surface to calculate the total precipitation rate for each simulation, shown in Figure 14. In Harris et al. (2021), the rate ranged from (5.6–26) × 10^24^ ions/s: while in this study, the rate ranges from (1.5–3.3) × 10^24^ ions/s for the simulations with low magnetospheric plasma density and (5.2–15) × 10^24^ ions/s for the simulations with high plasma density. Harris et al. (2021) showed that the precipitation rate increased linearly with the density of the magnetospheric plasma. Consistent with that result, we find that for all atmosphere cases the precipitation rate increases with the magnetospheric plasma density. For both the high and low density simulations, the precipitation rate drops quickly as the atmosphere column density increases to 0.5 × 10^15^ cm^−2^, and for simulations with higher atmosphere column densities the precipitation rate is approximately constant, leveling off at 2 × 10^24^ ions/s for simulations with low magnetospheric plasma density and 6.4 × 10^24^ ions/s for simulations with high magnetospheric plasma density.

## 5 Conclusions

To better understand how variations in Europa's atmosphere affect the bulk plasma properties and magnetic fields of Europa's plasma interaction, we conducted a parameter study that explores a reasonable parameter space for Europa's O_2_ atmosphere. Our design for the study was informed by the current best constraints on the atmosphere provided by analysis of HST observations (Hall et al., 1995, 1998; Roth et al., 2016) as well as predictions based on various models for the atmosphere (e.g., Cassidy et al., 2007, 2013; Teolis et al., 2017; and other references in Plainaki et al., 2018).

Our previous study by Harris et al. (2021) explored the range of variation in external magnetospheric plasma that Europa experiences within Jupiter's magnetosphere with a fixed neutral atmosphere throughout the study. In this work, we explored the effects of reasonable variations in Europa's neutral atmosphere on the plasma interaction and observed a larger variation in the ionosphere density. Each study explored the range of variation in the respective parameters that can be expected based on the current observations. This indicates that variation in Europa's neutral atmosphere could potentially have more significant effects on the density of Europa's ionosphere than variation of magnetospheric conditions. Based on the results of this study, the variation in Europa's ionospheric density observed by *Galileo* radio occultation experiments shown by McGrath et al. (2009) could be explained by variations in the density of Europa's atmosphere. However, we note that the variation of magnetospheric conditions explored by Harris et al. (2021) is relatively better understood than the variations in atmosphere parameters explored here. In particular, the magnetospheric magnetic field and plasma properties are known to vary periodically as Jupiter's dense plasma sheet wobbles up and down over Europa due to the tilt of Jupiter's internal dipole. The effects of this variation on the magnetic fields of the plasma interaction were observed in the *Galileo* data sets (Kivelson et al., 1999). While mechanisms that cause variation in Europa's atmosphere have been proposed through investigation with atmospheric models (Oza et al., 2019; Plainaki et al., 2013) and based on observations from the HST (Roth et al., 2016), the current limitations on observations of Europa's atmosphere prevent detailed measurement of possible time variation in the atmosphere density and spatial extent. Therefore, while our study shows that variations in the atmosphere could have a stronger effect on Europa's ionosphere than variations in magnetospheric parameters, it is not known whether these variations regularly occur in the way modeled here. Nevertheless, the qualitative similarities between the modeled and measured electron density profiles indicate that this is possible (compare panels in Figure 9).

In general, we found that as the column density of the atmosphere increased, the column density of the ionosphere and the pile up of magnetic field upstream of the moon increased as well. This caused simulations with atmospheres with higher column densities, either due to increased O_2_ surface density or increased scale height, to exhibit less precipitation of magnetospheric plasma. As the minimum atmosphere column density increased from ∼10^14^ cm^−2^ to 2.5 × 10^14^ cm^−2^ the total amount of precipitating magnetospheric plasma decreased sharply; at higher column densities the precipitation rate appears to saturate at 2 × 10^24^ ions/s for the simulations with low magnetospheric plasma density and 6.4 × 10^24^ ions/s for simulations with high plasma density. This behavior is controlled principally by the diversion of impinging plasma to the flanks of the interaction region by Europa's ionosphere.

The leveling‐off of the precipitation rate with increasing column density, combined with the effect observed by Harris et al. (2021), where the magnetospheric plasma precipitation rate increased approximately linearly with the magnetospheric plasma density, creates a more complete picture of how these two effects can alter the precipitation of magnetospheric plasma onto Europa's surface, and control the thermal plasma contribution to sputtering. Based on the results of Harris et al. (2021), the thermally sputtered contribution of atmospheric O_2_ may increase when Europa is near the center of Jupiter's plasma sheet, and decrease as the plasma sheet moves away and Europa is subjected to less dense magnetospheric plasma due to Jupiter's rotation. If the atmosphere becomes sufficiently dense, the results of this study suggest that the thermally sputtered contribution to the atmosphere will decrease. This coupling would tend to have a self‐limiting effect on increases in the density of the ionosphere: high ionosphere density would increase the pile‐up of magnetic field, reducing the sputtering yields from magnetospheric plasma (Figure 14) and energetic particles (Nordheim et al., 2022), leading to decreased sputtering contributions to the density of the atmosphere, and therefore reducing the amount of neutral O_2_ available to be ionized to form Europa's ionosphere. If the sources of mass for the ionospheric plasma are suppressed relative to the losses caused by recombination and the transport of plasma downstream, the ionosphere density would then decrease.

We also observed that the temperature of the precipitating plasma decreased significantly with increasing atmosphere column density, as shown by the orange contours in Figures 11 and 12. The temperature of the precipitating plasma generally decreased as the surface density of the atmosphere increased, and the simulations with the largest scale heights saw no plasma precipitate with temperatures higher than 100 eV. Because sputtering yields depend on temperature, this indicates that sputtering by thermal ions should be less efficient when the atmosphere is very dense or extended.

We simplified the input parameters for the simulations with the following two assumptions for the magnetic fields: we aligned the magnetospheric magnetic field with the Z‐EPhiO axis, and we did not include Europa's induced field. Doing so permitted us to focus on the interaction between the atmosphere and the plasma fluids without the obfuscation of additional asymmetries caused by the *B*_*X*_ and *B*_*Y*_ components of the background magnetic field. We expect that the main effect of including these components would be to tilt the interaction and cause the precipitation of plasma to be displaced elsewhere on Europa's surface, but should not significantly affect the total precipitation rate. More significant effects could be caused by the inclusion of the induced field, which is variable in strength and direction depending on the background Jovian field, and could contribute to shielding parts of the surface from direct precipitation (as was observed for energetic particles by Nordheim et al. (2022)).

In this work, we have primarily considered the effects of different states of Europa's atmosphere on the plasma interaction, and in turn how these affect the precipitation of magnetospheric plasma to the surface. In the previous study, Harris et al. (2021) examined the effects of variation in Jupiter's magnetospheric plasma on the plasma interaction and the subsequent precipitation. However, as we have noted above the precipitation of thermal plasma onto Europa's surface sputters off neutral particles that form Europa's atmosphere, implying the potential for feedback between Europa's atmosphere and plasma interaction. A complete simulation of this cycle is beyond the scope of the present study, requiring estimation of the neutral sputtering yields based on the precipitation of thermal plasma and coupling with a second model to update the steady state of the atmosphere based on this new information. Addison et al. (2022) have undertaken part of this process by determining the precipitation of thermal plasma onto Europa's surface based on hybrid modeling results, and subsequently calculating the sputtering yields based on the flux of plasma and the angles of impact with the surface.

Europa Clipper, NASA's upcoming flagship mission to Europa (Howell & Pappalardo, 2020), will conduct more than 40 passes near Europa's surface, collecting new in situ data on the plasma interaction. The simultaneous data sets collected by the Europa Clipper Magnetometer, the Plasma Instrument for Magnetic Sounding, and the MAss SPectrometer for Planetary EXploration/Europa (MASPEX) will remove uncertainty in the structure of Europa's atmosphere and its relationship with the local plasma populations and electromagnetic fields. Furthermore, measurements by the Ultraviolet Spectrograph (Europa‐UVS) will constrain the global structure of Europa's atmosphere and the normal range of its variability. These data sets will provide invaluable inputs for simulations such as those described in this work, and with improved inputs such simulations will better be able to characterize the plasma interaction by more accurately modeling the coupling between Europa and Jupiter's magnetosphere.

## Data Availability

The BATS‐R‐US code is publicly available for download as a component of the Space Weather Modeling Framework from the Center for Space Environment Modeling at the University of Michigan (https://clasp.engin.umich.edu/research/theory-computational-methods/swmf-downloadable-software/).
